# Politics and Sexual Difference

**DOI:** 10.3389/fpsyg.2019.02291

**Published:** 2019-10-18

**Authors:** Olivier Ouvry

**Affiliations:** Laboratoire UTRPP, Université Paris-Sorbonne-Cité, Villetaneuse, France

**Keywords:** feminism, capitalism, neoliberalism, sexual difference, sterility, radicalization

Our reflection begins with an article on Iran called “Les Iraniennes ne désarment pas”^1^, published in the French paper *Monde Diplomatique*. It highlights how Iranian women want to reclaim^2^ a place in society and precipitate a redistribution of power from a culture which has been male-dominated since the Khomeiny Revolution. Unfortunately, in Iran politics are controlled by the laws of religion, which excludes any po*s*sibility of parity between sexes. The resulting social tension is palpable.

What is remarkable in this article—although implicitly—is how Iran's political agenda today is a reaction to this feminist movement, which is itself a response to an overabundance of masculine power and growing phallicism, under many different forms. Hence, women dressed in chadors, the regression toward fundamentalism (the effects of which are evident in the relationship between men and women), the use of war and terror (both in internal and external policies) to maintain power, are secondary manifestations of the power dynamics between the two sexes. The issues here are not simply religious, and the attempt to explain them via religion or tradition is a form of rationalization.

We propose that the issues between the sexes are expressed in the political sphere—and apply this hypothesis to our western societies. What can we observe in France, for example, that would shed light on the relation between political struggles and the relationship between the sexes?

## Feminism and Financial Capitalism

The feminist movement in France achieved indisputable progress—such as the official parity between men and women in terms of legal rights (and before factual reality). This was not accomplished without conflict—militant actions were directed against the androcentric power established since the early nineteenth century^3^—and progress is fairly recent. Such progress is a result of political engagement within the feminist movement beginning in the 1970s/80s.

At the same time, a new economical ideology has emerged, characterized by the conversion of capitalism into neoliberalism, that is, into unbridled capitalism (also known as the financialization of economy^4^).

Do these discourses—feminist and neoliberal—reflect each other much like a mirror, just like feminism and fundamentalism in Iran? The historical concomitance of their rise in the West reinforces this hypothesis^5^. In this way we observe that the more one of these two discourses is reinforced, the more the other gradually rises, showing a reciprocity that evokes the mirror game, more precisely the specular one.

One of the unintentional side effects of this mirror like game is the universalization of people: on the feminist end is the demand for parity and equality between the sexes, that is of people's universalism^6^, and on the neoliberal, a universalization of people and companies^7^, all of which become either consumers or the objects of consumption. Both sexual desire and individual subjectivity are reduced so no individual appears to be different, either on a sexual or on social level. This absence of difference transforms individuals into consumers, forced to adapt to the virtues of exchange, goods and people (cf. new family configurations^8^, transgender issues^9^). The mission of companies is to give these consumers products that, rather than appeal to their desire, make them, through displacement, the objects of desire.

The paradox is that the asymptotic rise of these two discourses cause them to converge toward an unexpected radicality: a neutralization of difference, both on the level of desire and sexuality. Feminism and neoliberalism lead, in their asymptote, to an eradication of sexual difference both in conjugal and parental levels. Individuals are required to adhere to the uniformity of the consumer and working status. The disappearance of the subject of desire in the social sphere (for the benefit of the consumer) answers, concomitantly, to the neutralization of what constitutes difference in the conjugal field (the neutralization of sexual difference).

## Social Symptom

Such radicalization has consequences, and we identify two manifestations:

- Those concerning the conjugal field, where we find frigidity and fertility problems^10^.
- Those expressed in the social field, in two different ways:
  - As a sexual *in-difference* and a special attention to new forms of *jouissance*, such as new family configurations and *queer* expressions^11^.
  - As the radicalized expression of a struggling phallicism: less of an Islamic drift (punctual in our society) and more of a warmongering tone seen in certain discourses such as, in France, those of Bernard Henri-Lévi or Patrick Zemmour and appealing toward growing militarization and police constraints that menace individual rights—and even the voting tendencies of many countries (USA, Brazil, Eastern Europe…).

The first of these manifestations seem to us a consequence of the neutralization of sexual difference. The access to the Other's *jouissance*^12^ which specifies what a woman is, is indeed disturbed, and a female, phallic drift is the result^13^. Our hypothesis is that the consequences of such a neutralized social universal (the universality of the consumer status and the neutralization of the differences between the sexes) hinder the discovery of the subjective issues generated by puberty and the discovery of the *Feminine*. Hence, teenage girls remain tied to femininity—mirroring man's position, which is made of artifice and seduction (and is the position of the girl during childhood)^14^.

The second manifestation is the defensive, egoic, radical expression: either adhering (being *queer)* or opposing them in a specular game (radicalization). Both seem to us a reaction to the loss of the *Feminine* we evoke. They bewilder men with the emptying of the phallic function's support: its distinction with the Other *jouissance*. The logical response is radicalism.

In any case it is urgent to listen to such phenomena before (Lacan, 2002) taking the risk that they might crystallize. They manifest the shaking of our phallocentric society, thus proved precarious. Both have that social symptomatic value.

It is remarkable how some women find satisfaction in certain defensive positions, which are nevertheless poorly compatible with their access to desire—and have consequences to their fertility and ovulation aptitudes. It is important to highlight how their object-of-seduction position is indeed compliant to what liberal society prescribes^15^: presenting oneself as an object of consumption, styled according to the rules of the phallocentric, masculine predatory drive (the white cis male in his caricature^16^). But as we convoke only women to this place, what has become of their access to the Other *jouissance* that has become alien to them? The fierce struggle of narcissisms for a sexual undifferentiation is often the correlate, as we can see through the functioning of many of today's couples.

## Conclusion

The theory we extracted from the article on Iran proves to be pertinent, and can be applied to other social, religious and political configurations. Thus, an attempt to understand a country's political issues (both internal and foreign) could benefit from the analysis we have developed of today's sexual difference's inscription modalities in Iran.

Is this a new paradigm in the study of societies' history and organization? A more serious exploration of what we have identified as “social symptom” could lead to further discoveries.

## Author Contributions

The author confirms being the sole contributor of this work and has approved it for publication.

### Conflict of Interest

The author declares that the research was conducted in the absence of any commercial or financial relationships that could be construed as a potential conflict of interest.

## Footnotes

^1^Beaugé (2016).“Les Iraniennes ne désarment pas.” *Le Monde Diplomatique*, February 2016. This title could be translated as “Iranian women don't disarm.”

^2^Actually, Iranian feminism has existed since the 1950s and continues to persist despite theocratic power. Under the Shah, women were somewhat emancipated.

^3^Laqueur (1992).

^4^By unbridled capitalism we make reference to the contemporary evolution (since the 1980s) of capitalism toward a growing financialization of economy, the circulation of capital disconnected from the reality of industrial production.

^5^“What lies behind this shift is a sea-change in the character of capitalism. The state-managed capitalism of the post-war era has given way to a new form of capitalism – “disorganized”, globalizing, neoliberal. Second-wave feminism emerged as a critique of the first but has become the handmaiden of the second” (Fraser, 2013).

^6^Radical feminism seeks to free itself from gender differentiation (Cameron and Scanlon, 2010).

^7^Cf. the links between abstract universalism and liberal ideology (Rosenvallon, 2011; Gauchet, 2014).

^8^We refer to all new “becoming a parent” configurations: homosexual couples, single parenting, ART, surrogacy.

^9^“Homosexuality and heterosexuality, intersexuality and transsexuality do not exist outside of a colonial and capitalist epistemology (…) It is capital and not life which is reproduced” (Preciado, 2019, p. 27).

^10^As we hear them, in clinical and amicable settings.

^11^“It appears that queer is thus not attempting to seek liberation from the system of gender difference, but simply to take liberties with it” (Cameron and Scanlon, 2010).

^12^Freud insisted on a change in erogenous zones, from the clitoris to the vagina, in order to qualify what was new in puberty. Hence, pubertal novelty can be considered the advent of the Feminine (of what is absent in childhood), a pubertal real which cannot be inscribed in the phallic order. This *Feminine* corresponds to the Other sex, since it has no infantile phallic inscription and opens to the Other's *jouissance*, specific of women (Ouvry, 2001, 2018).

^13^In conformity with the capitalist project of universalization of consumers, that impedes the female position: a one-by-one position, *not-all* (Lacan, 1998).

^14^“…the alliances and collusions between “post-feminism” and neoliberalism are central to the construction of young women as ideal neoliberal subjects” (Rutherford, 2018, p. 91).

^15^McGowan (2016).

^16^“In championing neutrality as a liberating and democratic forces, liberalism is bound to deny the importance of sexual difference” (Hellerich, 2018, p. 4).
